# DLI-IT: a deep learning approach to drug label identification through image and text embedding

**DOI:** 10.1186/s12911-020-1078-3

**Published:** 2020-04-15

**Authors:** Xiangwen Liu, Joe Meehan, Weida Tong, Leihong Wu, Xiaowei Xu, Joshua Xu

**Affiliations:** 1 FDA/National Center for Toxicological Research, 3900 NCTR Rd, Jefferson, AR 72079 USA; 2 University of Arkansas at Little Rock, 2801 S. University Ave, Little Rock, AR 72204 USA

**Keywords:** Deep learning, Pharmaceutical packaging, Neural network, Drug labeling, Opioid drug, Semantic similarity, Similarity identification, Image recognition, Scene text detection, Daily-med

## Abstract

**Background:**

Drug label, or packaging insert play a significant role in all the operations from production through drug distribution channels to the end consumer. Image of the label also called Display Panel or label could be used to identify illegal, illicit, unapproved and potentially dangerous drugs. Due to the time-consuming process and high labor cost of investigation, an artificial intelligence-based deep learning model is necessary for fast and accurate identification of the drugs.

**Methods:**

In addition to image-based identification technology, we take advantages of rich text information on the pharmaceutical package insert of drug label images. In this study, we developed the Drug Label Identification through Image and Text embedding model (DLI-IT) to model text-based patterns of historical data for detection of suspicious drugs. In DLI-IT, we first trained a Connectionist Text Proposal Network (CTPN) to crop the raw image into sub-images based on the text. The texts from the cropped sub-images are recognized independently through the Tesseract OCR Engine and combined as one document for each raw image. Finally, we applied universal sentence embedding to transform these documents into vectors and find the most similar reference images to the test image through the cosine similarity.

**Results:**

We trained the DLI-IT model on 1749 opioid and 2365 non-opioid drug label images. The model was then tested on 300 external opioid drug label images, the result demonstrated our model achieves up-to 88% of the precision in drug label identification, which outperforms previous image-based or text-based identification method by up-to 35% improvement.

**Conclusion:**

To conclude, by combining Image and Text embedding analysis under deep learning framework, our DLI-IT approach achieved a competitive performance in advancing drug label identification.

## Background

### Motivation

Drug control and drug distribution play a significant role in providing consumers and health professionals with the products that they need. Illegal, illicit, unapproved, counterfeit, and potentially dangerous drugs can cause severe harm to patients as well as the healthcare providers. The effectiveness of the drug control system depends on adherence to policies (broad, general statements of philosophy) and procedures (detailed guidelines for implementing policy). The importance of an up-to-date policy and procedure manual for drug control can-not be overestimated [1]. However, the investigation process can take time, practically when rejecting a questionable drug product or supplier.

To accelerate the process of investigation, the institute must establish and maintain an adequate record in the reference dataset containing images provided by investigators of previously rejected drugs. When a drug is under suspension, it could be evaluated by retrieving drug images from a historical reference dataset for the most similar candidates. Admissibility would be provided to an investigator for determination without examination and investigation if at least one of the candidates exactly matched the testing drug image. The goal of our model is to accelerate the processing time by using recognized text for retrieval instead of traditional input from a keyboard. Investigators would only need to take an image of a drug’s pharmaceutical packaging and our model would automatically extract text from the image and return the most similar candidates from the historical reference dataset. Finally, the investigator could make a quick decision for the necessity of investigation.

We obtained an internal image dataset of previously rejected drugs, and developed image-based and text-based methods to identify if two label images are for the same drug. In current stage, only image and no other extra information (such as meta-data documents) of the rejected drugs would be involved for the analysis. Utilizing the rich text information from images for similarity analysis, this paper answers three important questions: First, how to detect and recognize text in images taken by investigators? Second, how to retrieve the most similar candidates from a reference dataset? Third, does this method have a better performance rate than the standard image similarity analysis? These questions will be answered in the methodology and result sections of our paper.

### Related work

#### Image-based similarity analysis

Content-Based Image Retrieval System (CBIR) is a method of retrieving an image based on the input image. In CBIR, the content of an image is analyzed in terms of attributes such as colors, shapes, and textures of the image. Perceptual Hash (Phash) is mostly used for similarity identification. Phash is a hashing function which can be used in Crypto-hashing, Digital Watermarking, and Digital Signal Processing. There are four types of Phash algorithms currently in use: (1) DCT (Discrete Cosine Transform) based Hash; (2) Marr-Hildreth Operator based Hash; (3) Radial Variance based Hash; (4) Block Mean Value based Hash. In this paper, we complete experiments using Average Perceptual Hashing Algorithm, which is like Block Mean Value based Hashing Algorithm [2] [3]. There are many drawbacks to only relying on image-based similarity analysis, such as computational pressure on the pixel-based comparison and significant feature engineering prior to training. Additionally, the result is more sensitive to environment changes, different resolutions, non-uniform illumination, and partial occlusion. For instance, a drug label with the same text but a different background image would likely result in a low similarity score using image-based similarity analysis, but the text-based similarity score would be higher, and the result would be more accurate.

#### Deep learning-based image retrieval methods

Recently, many image retrieval methods emerged with the revolutionary of deep learning. Learning fine-grained image similarity with deep ranking, the model employs deep learning techniques to learn similarity metric directly from images [4]. Deep image retrieval: Learning global representations for image search is another method, which could produce a global and compact fixed-length representation for each image by aggregating many region-wise descriptors [5]. But these methods are not applicable to our case since our datasets are limited and we don’t have sampled triplets (Q, A, B) for training. There is also content-based image retrieval solution such as [6] proposed, but it is difficult to apply in our project because of our datasets are short of pairs of images for metric learning.

#### Text detection and recognition

Two steps are necessary to extract the text from images: text detection and text recognition. Since Optical Character Recognition (OCR) engine is only suitable for recognizing text from images with uniform backgrounds, we added another step, Scene Text Detection and Recognition (STDR), before OCR engine. The cropped sub-images with uniform backgrounds was then ready for recognition with for OCR engine. In our experiments, the popular deep neural network Connectionist Text Proposal Network (CTPN) was trained and applied to detect text from images. The architecture of CTPN network is displayed in Fig. 2b.

Traditional OCR engines can extract text from image; however, these methods are only suitable for a unique and simple background. Images in our dataset have diverse backgrounds with a variety of text font and color. OCR systems are widely used to convert images of typed, handwritten, or printed text into machine-encoded text. There are dozens of commercialized OCR systems for text recognition, such as CIB OCR, ABBYY, and Asprise OCR. In this paper, we utilize the Tesseract OCR Engine from Google, which is an open source software for various operating systems. Simplified architecture of Tesseract is displayed in Fig. 2c. In 2006, it was considered one of the most accurate open-source OCR engines available [7]. It has been sourced by HP since 1985 and developed by Google since 2006. It is trained by typed printed text on about 400,000 text-lines spanning about 4500 fonts in 130 languages. Tesseract 4 deployed a recurrent neural network model (LSTM) based OCR engine, which focuses on text line recognition. Tesseract supports Unicode (UTF-8) and can recognize more than 100 languages “out of the box” [8].

#### Word embedding and sentence embedding

Distributional representation is based on the hypothesis that linguistic terms with similar distributions have similar meanings. These methods usually take advantage of the co-occurrence and context information of words and documents, and each dimension of the document vector usually represents a specific semantic meaning [9]. Due to the issues of meaning ambiguity and vector sparsity, distributional representation has a limited performance on text similarity analysis. Hence, distributed representation is utilized in deep learning research on Natural Language Processing (NLP), which converts data into vectors. Once data are converted into vectors, we can evaluate the similarity by calculating the distance between vectors. Deep neural network models like Word2vec [10], GloVe [11], ELMO [12], and BERT [13], transform words in vectors through training language models using billions of documents from Wikipedia, news articles, and webpages worldwide.

The universal sentence encoder is suitable for our task because it is a transfer learning model for NLP tasks, which presents a challenge for data hungry deep learning methods. We can transfer features, which are encoded in vectors trained from huge natural language datasets, to our drug text dataset. Many models have transferred features through pre-trained word embedding such as those produced by word2vec [10] or GloVe [11]. However, these vectors have difficulty solving our problems due to noise, deviation, and incorrect recognitions. There is also a novel word embedding called BioBERT [14]. However, applying this is not that straight forward. In fact, based on our internal preliminary result, it is surprisingly that Google Sentence Encoder showed quite competitive performance to BERT and BioBERT, if not better. The underlying reasons we can think of are: (1) the drug labeling may be more similar to general text document rather than scientific articles (such as PubMed). (2) It is tricky to get the embedding vector of the whole sentence from all its words, as we also observed that a simple average value would exaggerate the weight of trivial words (there are a lot in labeling imprints) in the sentence.

## Methods

### Datasets

The image samples were collected from Daily-Med; 43% of the images belonged to opioid drugs label and 57% belonged to non-opioid drug labels. The images are public at Daily-Med website for downloading: https://dailymed.nlm.nih.gov/dailymed/spl-resources.cfm. Table 1 is the distribution of images among the drugs. One drug label may contain multiple images. All drug labeling were identified by “SET-ID” so images having the same “SET-ID” are identified as the same drug label. Following is the list of Established Pharmacologic Class (EPC) of Opioid drugs in query: Opioid Agonist [EPC], Opioid Agonist/Antagonist [EPC], Opioid Antagonist [EPC], Partial Opioid Agonist [EPC], Partial Opioid Agonist/Antagonist [EPC], and mu-Opioid Receptor Agonist [EPC].

**Table 1:** Distribution of images in drug label

DRUGS	Number of image samples per drug label	Number of unique labels	Total images
Opioid Drugs	2	196	392
	3	148	444
	4	80	320
	5	42	210
	6	25	150
	7	19	133
	8	9	72
	9	2	18
	10	3	30
Non-opioid Drugs	5	473	2365

### Text detection

**Fig. 1:**
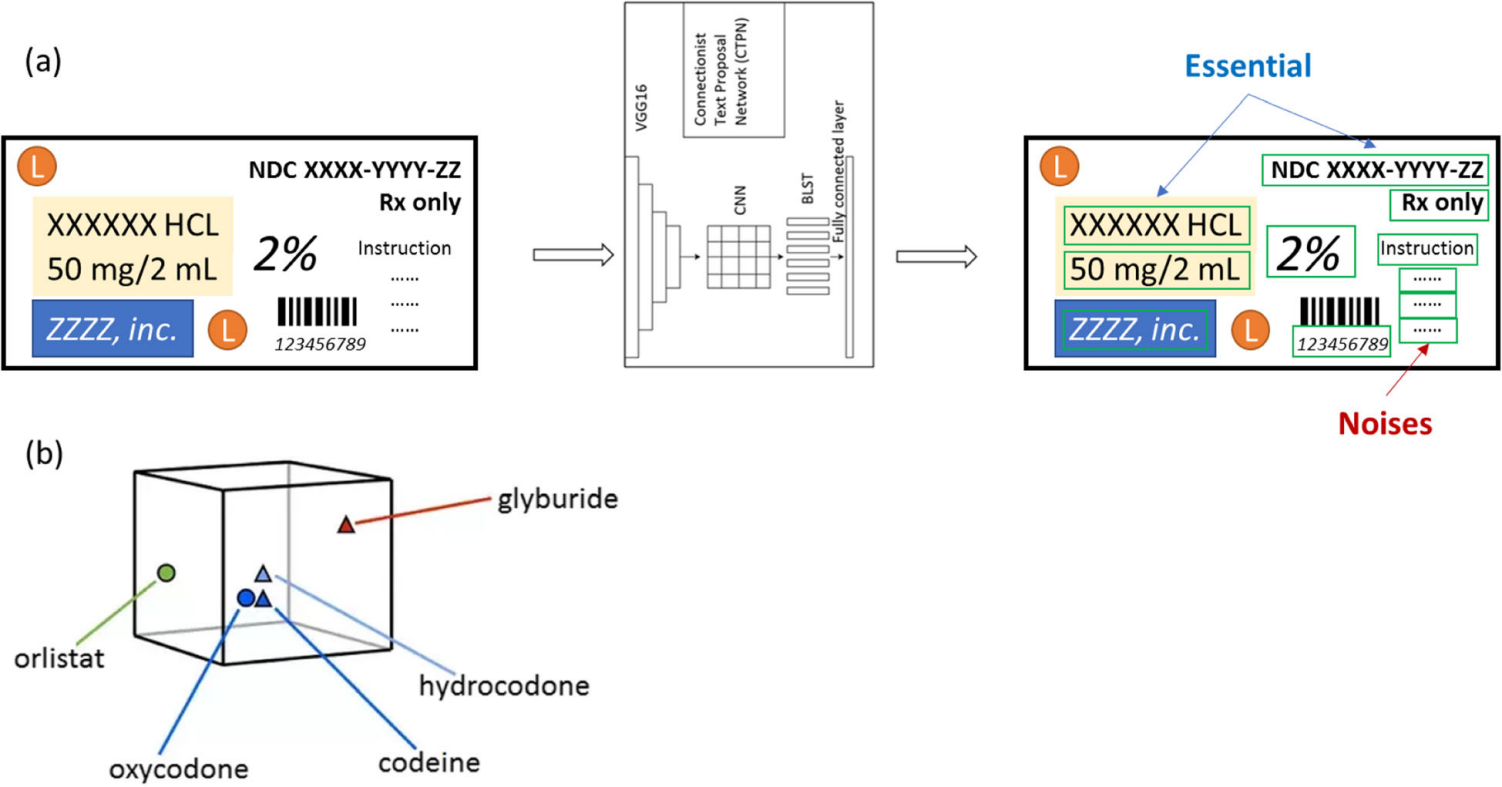
(**a**) Sample result of text detection by CTPN. Some texts detected by CTPN may be noises for later drug product recognition. (**b**) One-word embedding example “codeine”. As shown, the corresponding vector of “codeine” is closer to “oxycodone” and “hydrocodone”, while vector of “codeine” is further from “glyburide” and “orlistat”, based on the functionality difference of drugs

Images in our dataset had diversity in text font, color, scale, and orientation. Some images even had a very complex background. Moreover, other interference factors existed such as noise, distortion, low resolution, non-uniform illumination, and partial occlusion. After researching several text detection algorithms [15–17], Connectionist Text Proposal Network (CTPN) [18] was chosen as the first step of our model to detect text from images. The CTPN detects a text line in a sequence of fine-scale text proposals directly in convolutional feature maps [18]. It is an efficient end-to-end text detector. Following Fig. 1a is an example result of text detection by CTPN. As Fig. 1a shows, VGG16 [19] model is followed by the convolutional neural network (CNN). The sequential windows in each row are recurrently connected by a Bi-directional LSTM (BLSTM) [20]. The model was developed via Tensorflow. We trained the model on ICDAR 2015 benchmarks [21] on a Linux machine with Nvidia TITAN X GPU card for 1 week. Then, the trained model was used to detect text in our Non-Opioid drug and Opioid drug images.

### Levenshtein distance for text similarity

Levenshtein distance, also known as edit distance, is widely used for quantifying the dissimilarity of two text strings. This algorithm calculates the minimum amount of operations transforming a text string into another string, that includes replacement, deletion, and insertion [22] –[23]. For example, an optimal way to compare the two strings ‘MONDAY’ and ‘SATURDAY’, is to insert letters “S” and “A” and substitute “M”, “O” and “N” with “T”, “U” and “R”, respectively, leading towards a Generalized Levenshtein Distance (GLD) of 5 [24]. To handle noise and incorrect recognitions in text, we introduced Partial Levenshtein Distance (PLD) since PLD can outperform GLD, such as in the following text.

Text1: Methadone Hydrochloride Oral Solution USP, NDC 0054–3556-63.

Text2: Methadone Hydrochloride Oral Solution USP, 10 mg per 5 ml, keep in secure area and protected from diversion.

Text1 and Text2 are extracted from the same drug. The difference is that Text1 includes the name and NDC number and Text2 includes the name and additional noise text. With this example, we can see that the similarity score using these two methods is totally different: GLD: 55 and PLD: 76.

In our experiments, we combined GLD and PLD to reach the best Levenshtein distance result used as a baseline to compare our novel sentence embedding method.

### Semantic similarity analysis

To retrieve the most similar candidates from reference datasets, it is necessary to represent each text sentence recognized from the test drug image, and comparer the text similarity based on representation. So, the embedding process of a word as a vector enables calculating the similarity score via inner product when a single word is recognized. If the text recognized from drug images are multiple words with only essential information, such as drug name and manufacture, the average of words vectors is ready for calculating inner production.

Let *S* be the sentence extracted from the image, which is represented as:

$$ \mathcal{S}=\left\{{\mathcal{w}}_1,{\mathcal{w}}_2,\dots \kern0.5em {\mathcal{w}}_i,\dots {\mathcal{w}}_n\right\} $$

Where ${\mathcal{w}}_i$_*,*_ is the vector of ${\mathcal{i}}_{th}$ word in sentence, and *n* is the length of sentence (number of words). Then the final vector of sentence is represented as:

$$ \overline{\mathcal{w}}=\frac{\sum {\mathcal{w}}_i}{n} $$

Besides the drug name and manufacturer information, there is a bunch of information such as “Directions For Use”, “Caution”, “Address” and “Store instructions” are extracted which is noise for text similarity analysis. Even incorrect recognition due to a damaged image or detection and recognition limit could weaken the final vector of a sentence. Under these circumstances, the final average vector cannot represent the drug identity due to the dilution made by noise or the deviation made by the incorrect recognition result. Embedding a sentence along with words and the context of the whole sentence needs to be captured in that vector. Therefore, the use of a universal sentence encoder is proposed by Google Research [25].

The Universal Sentence Encoder encodes text into high dimensional vectors that can be used for text classification, semantic similarity, clustering, and other natural language tasks. The pre-trained Universal Sentence Encoder is publicly available in Tensorflow-hub. It comes with two variations, one trained with Transformer encoder and the other trained with Deep Averaging Network (DAN) [26]. The two have a trade-off of accuracy and computational resource requirement. While the one with Transformer encoder has higher accuracy, it is computationally more intensive. The one with DAN encoding is computationally less expensive and has slightly lower accuracy. The model trained with Transformer encoder was utilized in our experiment for higher accuracy. A one-word embedding example using word “codeine”, a common opioid drug, is shown in Fig. 1b.

All texts from images are recognized for similarity identification. Texts are represented as:

$$ \mathcal{T}=\left\{{\mathcal{t}}_1,{\mathcal{t}}_2,\dots {\mathcal{t}}_i,\dots {\mathcal{t}}_m\right\} $$

Where, m is the number of texts, ${\mathcal{t}}_i$ is a 512-dimensional vector representing each text from image.

Similarity score between two extracted texts $\mathcal{A}$ and $\mathcal{B}$ is calculated by cosine similarity:

$$ Similarity\left(\mathcal{A},\mathcal{B}\right)=\frac{\mathcal{A}\bullet \mathcal{B}}{\left\Vert \mathcal{A}\right\Vert \times \left\Vert \mathcal{B}\right\Vert }=\frac{\sum \limits_{\mathcal{i}=1}^{512}{\mathcal{A}}_{\mathcal{i}}\times {\mathcal{B}}_{\mathcal{i}}}{\sqrt{\sum_{\mathcal{i}=1}^{512}{\mathcal{A}}_{\mathcal{i}}^2}\times \sqrt{\sum_{\mathcal{i}=1}^{512}{\mathcal{B}}_{\mathcal{i}}^2}} $$

Where, $$ \mathcal{A}\in \mathcal{T}, and\ \mathcal{B}\in \mathcal{T} $$.

## Results

### Study framework

**Fig. 2:**
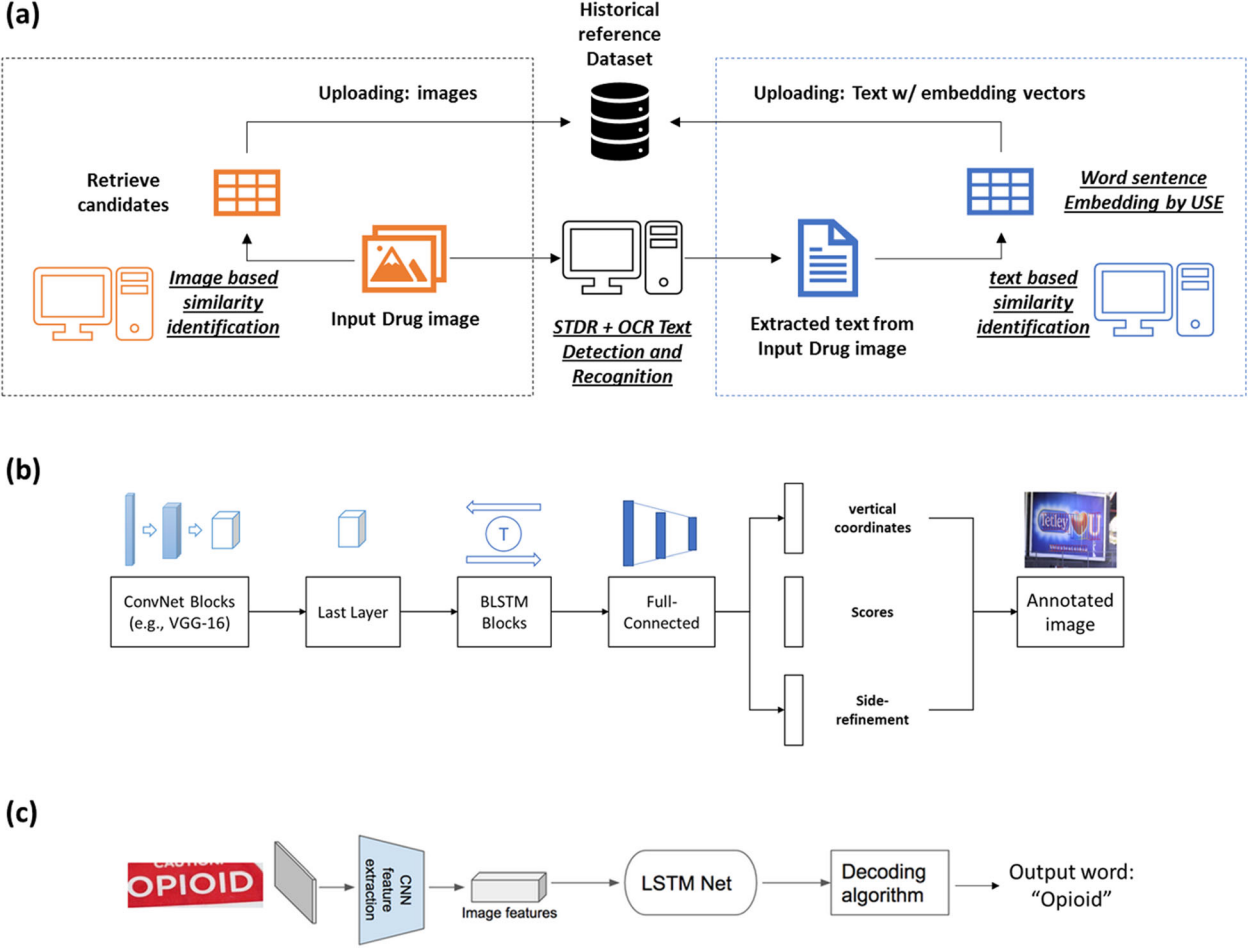
(**a**) The proposed drug label identification approach. (**b**) Architecture of the Connectionist Text Proposal Network (CTPN). (**c**) Simplified Architecture of Tesseract OCR

As we mentioned in the previous section, it is difficult to detect and recognize text from images using current methods. On the other hand, our sentence embedding based text similarity measure played a significant role retrieving the top N candidates from the reference dataset. Figure 2a displays the overview of the work flow as follows. Starting with the input drug image, we first used Scene Text Detection and Recognition (STDR, Fig. 2b**)** plus Optical Character Recognition (OCR, Fig. 2c) to detect and recognize embedded texts in the input image. Text based similarity identification was then applied to retrieve top-ranked candidate images from the historical reference dataset. On the other side, the image-based similarity identification was directly applied to the input drug image to retrieve top-ranked candidates from the historical reference dataset. Finally, these results were uploaded to the historical dataset to update the reference following human-level assessment and validation.

### Experiments

As mentioned before, we combined text detection model Connectionist Text Proposal Network (CTPN) [19] and Tesseract OCR Engine to extract text from drug labeling images. CTPN model is trained on the ICDAR 2015 benchmark [21] and the cropped image is provided to OCR Engine for recognition. To obtain the best performance in evaluating the sentence level similarity identification, recognized text results from Google Vision Cloud are utilized in all the experiments. Then, each text sentence was encoded to a 512-dimensional vector. Finally, the similarity scores were calculated with all texts in the reference dataset.

Two groups of experiments are conducted, the first was conducted on mixed images of opioid and non-opioid drug labeling, the second was conducted on images of opioid drug labeling. In each group, 300 images were randomly selected for the testing dataset, and the rest of the images were used as a reference dataset for retrieving the source. The number 300 is defined because, in all the experiments, the result was converged below 300 (around 280).

We completed four series of experiments: The first was a base line, an image-based similarity identification using the method of Average Phash. The second recognized text results followed by Levenshtein Distance for similarity identification. The third was our proposed method, which recognized text followed by the sentence embedding similarity identification. The forth was combined image-based and text-based similarity identification with equal weight, 0.5.

### Recognition results

Following Table 2 and Table 3 show image-based and text-based similarity identification evaluated on Recall @k and Accuracy @k. To optimize our text embedding and similarity identification result, we used Google Cloud Vision recognized text instead of STDR-OCR recognized text for embedding.

**Table 2:** Retrieval results on mixed images of Opioid and Non-Opioid drug label

	P @k						R @k					
Methods	k = 1	k = 2	k = 3	k = 4	k = 5	k = 6	k = 1	k = 2	k = 3	k = 4	k = 5	k = 6
Image-based	0.630	0.535	0.470	0.413	0.346	0.297	0.152	0.258	0.340	0.398	0.417	0.429
Levenshtein with text	0.480	0.550	0.480	0.425	0.376	0.333	0.116	0.265	0.347	0.410	0.453	0.482
Embedding of text	0.800	0.720	0.640	0.565	0.478	0.405	0.193	0.347	0.463	0.545	0.576	0.586
0.5 * Image + 0.5 * Text embedding ^†^	0.800	0.725	0.640	0.570	0.480	0.410	0.193	0.349	0.463	0.549	0.578	0.593
Improvement^*^	27%	32%	33%	34%	28%	23%	27%	32%	33%	34%	28%	23%

**Table 3:** Retrieval results on images of Opioid drug label

	P @k						R @k					
Methods	k = 1	k = 2	k = 3	k = 4	k = 5	k = 6	k = 1	k = 2	k = 3	k = 4	k = 5	k = 6
Image-based	0.650	0.540	0.453	0.395	0.342	0.302	0.193	0.320	0.404	0.469	0.507	0.537
Levenshtein with text	0.580	0.495	0.407	0.340	0.298	0.263	0.172	0.294	0.362	0.404	0.442	0.469
Embedding of text	0.800	0.665	0.560	0.495	0.436	0.388	0.237	0.395	0.499	0.588	0.647	0.691
0.5 * Image + 0.5 * Text embedding ^†^	0.88	0.755	0.633	0.568	0.510	0.460	0.261	0.448	0.564	0.674	0.757	0.819
Improvement^*^	35%	40%	40%	44%	49%	52%	35%	40%	40%	44%	49%	52%

*Definition of P @k and R @k:

Precision @ rank K (P@K) is proportion of retrieved drug labeling that have same label with test drug label,

$$ P@K=\frac{\left|\left\{ Drug\ labels\ same\  as\  test\ drug\right\}\cap \left\{ Retrieved\  top\ k\  labels\right\}\right|}{\left|\left\{ Retrieved\  top\ k\  labels\right\}\right|} $$

Recall @ rank K (R@K) is proportion of same drug labeling with test drug that are retrieved.

$$ R@K=\frac{\left|\left\{ Drug\ labels\ same\  as\  test\ drug\right\}\cap \left\{ Retrieved\  top\ k\  labels\right\}\right|}{\left|\left\{ Drug\ labels\ same\  as\  test\ drug\right\}\right|} $$

From the results, image-based similarity identification had a lower Recall and lower Accuracy due to sensitivity to the environment, image color, and text font. Our text-based similarity identification had both higher Recall and Accuracy. In addition, we conducted more experiments using combined image-based and text-based method, which lead to better results compared to using a text-based method only. Table 2 displays the results for the mixed data of opioid and non-opioid drug label images. A maximum 34% improvement was achieved on Recall @4 and Precision @4 by our novel method compared to the best result from traditional methods. Table 3 displays the results on opioid drug labeling, where maximum 52% improvement was achieved on Recall @6 and Precision @6 by our novel method compared to the best result of traditional methods. Based on these results, the best solution is combined image-based and text-based similarity identification method with equal weight.

* Improvement is achieved by comparing our result with best result of traditional methods: image-based method or Levenshtein distance for similarity analysis.

† 0.5 * Image + 0.5 * Text embedding is achieved through trying all weight combination from 0.1 to 0.9 by increasement 0.1.

## Discussion

**Fig. 3:**
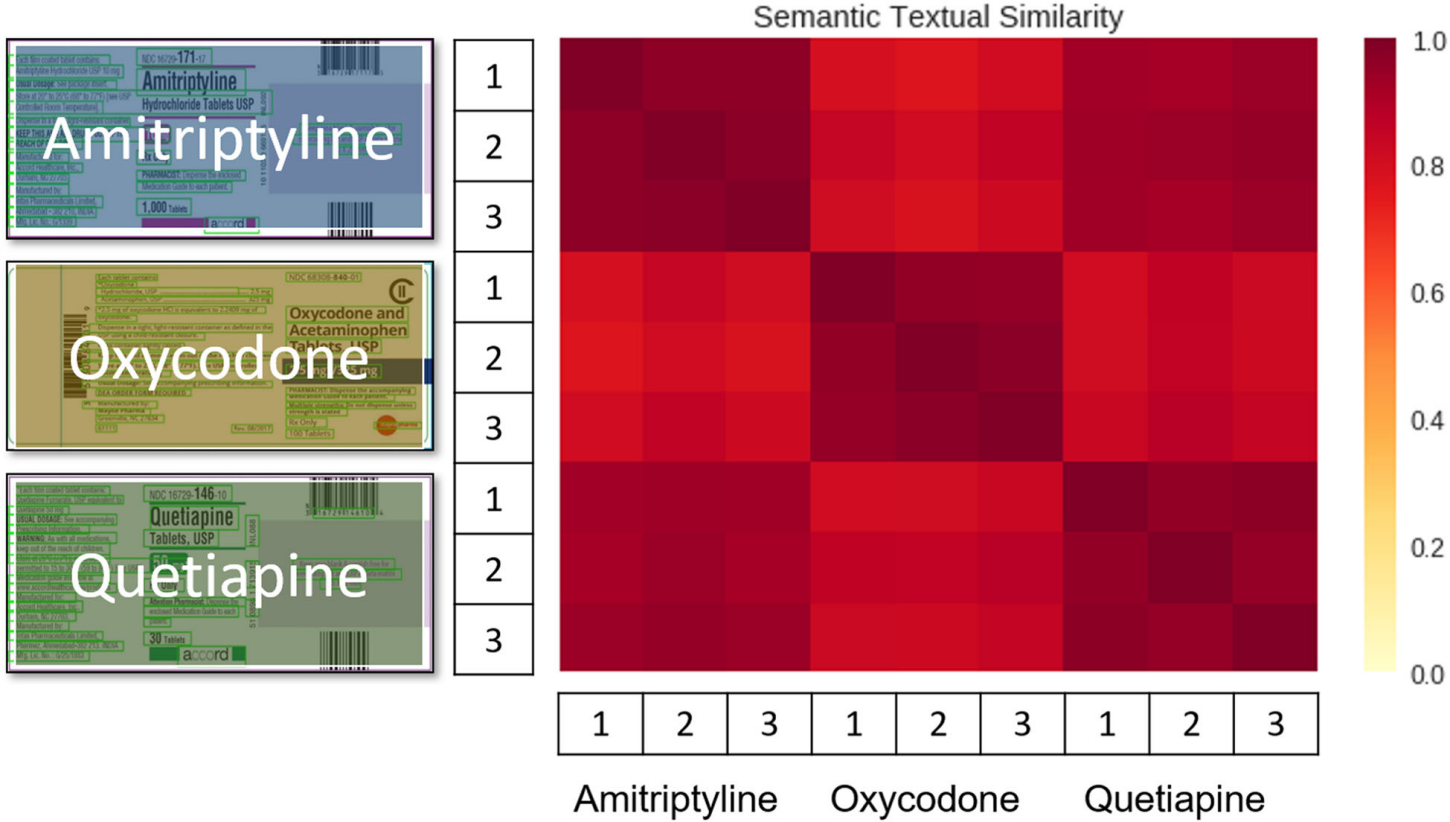
Similarity analysis among three opioid drug labeling. Each drug contains three distinct label images. Similarity scores range from 0 to 1 (most similar)

Text-based similarity identification with sentence embedding:

During our investigation we wondered why the text-based similarity identification with sentence embedding produced better results than image-based similarity identification? To further study this, we picked three drug labeling that each containing three drug images. Figure 3 shows the similarity matrix built from the three drug labels and nine texts, extracted from the nine images. It is reasonable that the text belonging to the same drug label has a darker color, since the similarity approaches 1. The interesting point is that text from the Amitriptyline drug label had a higher similarity to text from Quetiapine. This happened because sentence embedding encodes the most essential information on drug labeling, including the drug name as well as manufacturer information. In this test, the drug labeling of Amitriptyline and Quetiapine had the same manufacturer name.

**Fig. 4:**
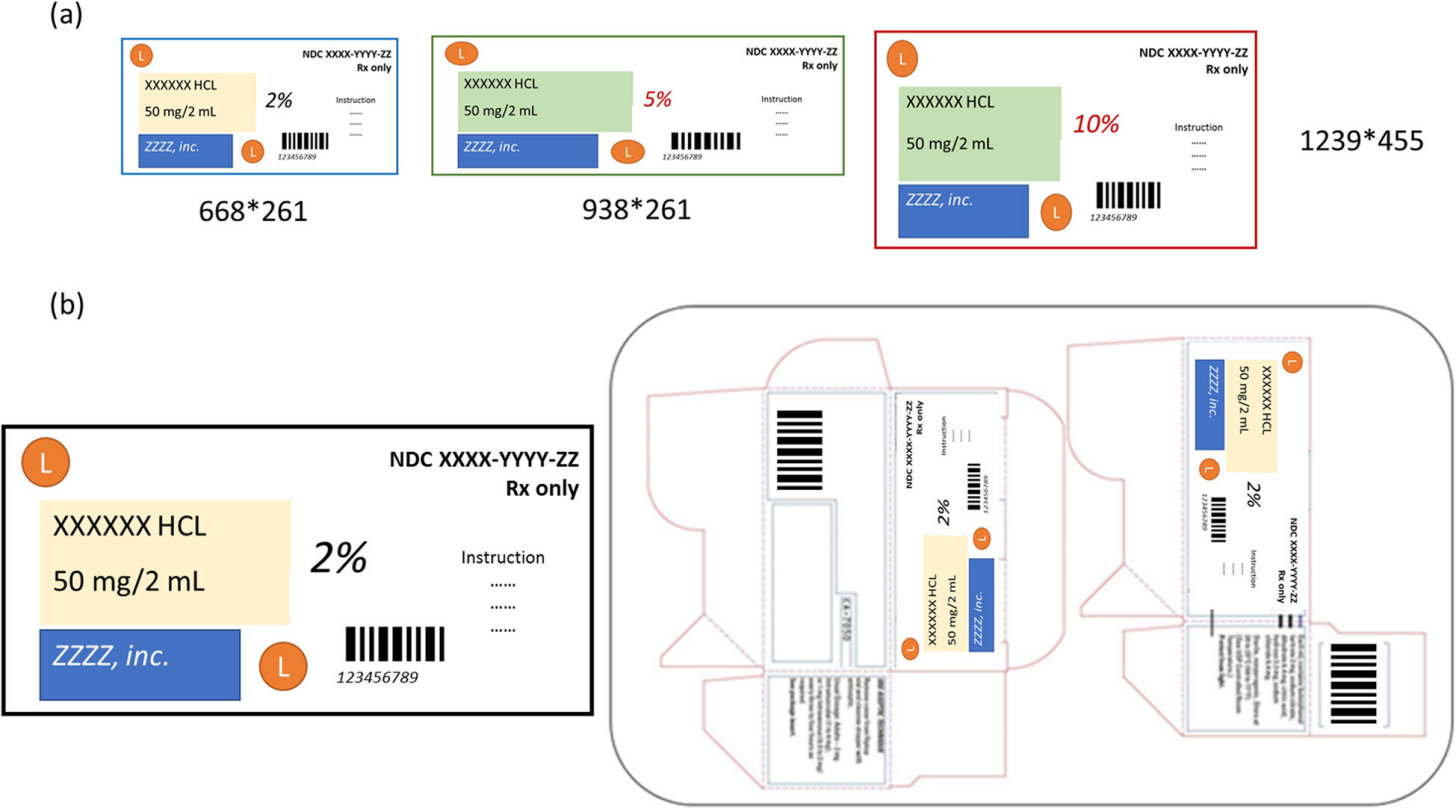
Advantages of our approach for regular image-based similarity analysis. (**a**) Example of different image resolutions for the same drug label. (**b**) Example of different images of the same drug label with stable text

Advantages to regular Image-based similarity identification:

As shown in this study, text-based method performs better then image-based similarity approach in images identification. Two potential reasons leading to the outperformance may be that text-based similarity identification is better in dealing with image resolution and for drug labeling objects, the content of text is more stable that image patterns. For instance, in Fig. 4a, when we changed the image resolution and used it as the new input (right image in pairs), Recall @6 was 0 for image-based similarity identification while text-based similarity identification using embedding was 3. Also, for the drug image in Fig. 4b, because the big difference of image while stability of text, Recall @6 was 0 for image-based similarity identification, while text-based similarity identification using embedding was 2.

## Conclusion

In this paper, we solved the challenging problem of identifying questionable drugs during drug distribution. With the help of our model, investigators can easily make a quick decision to accept or deny a drug based on top candidates, retrieved from a historical reference dataset. Our proposed method utilizes cutting-edge deep neural network and transferred features from Google’s universal sentence encoder, which was trained from billions of documents.

In addition to higher Recall @k and Precision @k results compared to image-based similarity identification, our method is more stable since the image-based method is sensitive to environment changed, different resolutions, non-uniform illumination, and partial occlusion. Additionally, extracted text can be easily used for database querying as well as for future online searches for drug-related information. In addition, the accurate text detection and recognition methods could serve for automatically image caption generation, to support further researches such as developing supervised model of auto description generation of drug labeling image, etc.

These accurate and efficient retrieval results also suggest that our proposed method is promising for other types of product similarity identification. Especially for products with rich text information on images.

## Data Availability

Drug labeling data used in this study is publicly available at FDALabel website: https://nctr-crs.fda.gov/fdalabel/ui/search and DailyMed: https://dailymed.nlm.nih.gov/dailymed/spl-resources.cfm.

## Abbreviations

BLSTM: Bi-directional LSTM
CNN: Convolutional Neural Network
CTPN: Connectionist Text Proposal Network
CBIR: Content-Based Image Retrieval System
DAN: Deep Averaging Network
EPC: Established Pharmacologic Class
GLD: Generalized Levenshtein Distance
LSTM: Long-Short Term Memory
NLP: Natural Language Processing
OCR: Optical Character Recognition
PLD: Partial Levenshtein Distance
STDR: Scene Text Detection and Recognition
